# Reframing Aging: Two-Spirit and Indigenous LGBTQ+ Perspectives on Living Well with HIV

**DOI:** 10.1093/geroni/igaf122.240

**Published:** 2025-12-31

**Authors:** Chase Bryer

**Affiliations:** Brown University School of Public Health, Providence, Rhode Island, United States

## Abstract

This study explored the intersections of social identities and aging experiences among Indigenous Two-Spirit (2S) and LGBTQ+ people living with HIV (PLWH). While advances in HIV treatment have increased their lifespan, these individuals still face high infection rates and health disparities due to intersectional stigma, discriminatory stress, and historical trauma. The primary researcher aimed to understand what it means to “age well” with HIV within this community, and how their cultural perspectives challenge conventional views of “successful aging”. Based on 15 oral life review interviews, the primary researcher used narrative analysis to understand how people make meaning of their life experiences by closely examining their narratives. Findings suggest that Indigenous cultural practices helped participants accept their HIV diagnoses and adhere to antiretroviral treatment (ART), while Indigenous cultural generativity—passing down cultural knowledge—was an important element in aging well with HIV. Notably, participants viewed HIV as a “gift” or “messenger,” which allowed them to realign their life purposes, especially through Two-Spirit activism and HIV advocacy. These insights emphasize the importance of integrating cultural perspectives into aging and HIV care for Indigenous 2S/LGBTQ+ individuals. The study provides valuable lessons for future research, policy, and healthcare practices that are strengths-based, and aimed at addressing health inequities in this community.

